# The Ability to Sustain Facial Expressions

**DOI:** 10.1097/SCS.0000000000010054

**Published:** 2024-02-16

**Authors:** Hilde Schutte, Freek Bielevelt, Hafsa Emohamadian, Marvick S.M. Muradin, Ronald L.A.W. Bleys, Antoine J.W.P. Rosenberg

**Affiliations:** * Department of Oral- and Maxillofacial Surgery, University Medical Center Utrecht, Utrecht; † Radboudumc 3D Lab, Radboud University Medical Centre, Nijmegen; ‡ Department of Functional Anatomy, University Medical Center Utrecht, Utrecht, The Netherlands

**Keywords:** Facial expression, facial muscles, image processing, computer-assisted, imaging, 3-dimensional, magnetic resonance imaging

## Abstract

To gain more insight into facial muscle function, imaging during action would be optimal. Magnetic resonance imaging is highly suitable for visualizing facial muscles. However, magnetic resonance imaging requires the individual to remain as still as possible for a while. Knowledge of the ability to sustain facial expressions is requisite before scanning individuals. This could help adapting the scanning protocol to obtain optimal quality of imaging the muscles in action. A study, including 10 healthy volunteers, was done to perceive the extent of movement while holding facial expressions of smiling and pouting. During 6 minutes, 3-dimensional photographs were taken every consecutive minute while the participants maintained their facial expressions as motionless as possible. The movement was objectified by creating distance maps between the 2 models and calculating the Root Mean Square using the software 3DMedX. The results showed that most movements occurred in the first minute, with a decrease of the intensity of the expression. After the first minute, the expression, although less intense, could be held stable. This implies that magnetic resonance imaging scanning during facial expression is possible, provided that the scanning starts after the first minute has elapsed. In addition, results demonstrated that more slackening of the muscles while smiling compared with pouting.

Facial expressions are fundamental nonverbal cues in human communication. They play a pivotal role in conveying emotions, intentions, and social interactions and form a universal communication system in society.^1^ These dynamic facial movements are produced by minute changes in the facial muscles. For example, when the corners of the mouth are pulled to the side and back, a broad smile forms on the face that implies friendliness, joy, and reassurance and conveys a sense of belonging.^2^ Although the facial muscles are small in size, they play a major role in conveying powerful messages. The analysis of facial expressions has been extensively studied because changes in facial expressions have a major impact on physical, psychological, and social well-being.^3^

Some operations are known to impede facial expressions. For example, the Le Fort I osteotomy is a procedure that is widely used to correct abnormalities in the midface.^4^ This surgical procedure can result in a reduced ability to smile broadly and may affect facial dynamics.^5–8^ Other situations where facial animation is altered are facial paralysis^3^ or orofacial clefts, even after surgical correction.^9^ A thorough understanding of the anatomy, physiology, and function of facial muscles is, therefore, crucial.

To get a better understanding of the anatomy and its correlation to muscle function, ideally, muscle activity should be captured while executing different facial expressions. This would give insight in, both, the anatomy and function of the facial muscles and would contribute significantly to the understanding of the correlations between the 2. Magnetic resonance imaging (MRI) is a very suitable imaging technique for analyzing facial muscles.^9–12^ During MRI scanning, subjects are typically instructed to remain as motionless as possible to minimize motion artifacts.^13^ However, the lengthy acquisition times required for high-quality MRI scans make subjects susceptible to unintended movements. These can degrade image quality and limit the accuracy of diagnostic interpretations. Especially while holding facial expressions, unintended movements might impede the imaging process, hindering the acquisition of clear and precise facial muscle data.

To date, no research has been conducted to investigate if MRI can capture facial expressions. Neither has it been explored how long an individual could sustain a certain facial expression. To address these questions, a preliminary investigation was conducted to assess the stability of 2 common facial expressions: smiling and pouting. These 2 expressions were chosen for their distinct facial muscle activation patterns, providing 2 extreme positions of the corners of the mouth. Moreover, these expressions are the most reproducible facial expressions, allowing us to explore their stability during prolonged MRI scanning.^14–17^ By investigating the stability of facial expressions during a prolonged period of time, this study looks into the feasibility of conducting MRI scans while holding facial expressions.

## METHODS

### Population and Data Acquisition

The local ethics committee approved this study (study number 22-068). This study was conducted in compliance with the World Medical Association Declaration of Helsinki on medical research ethics. For this study, 10 healthy volunteers, 5 males and 5 females, aged between 18 and 45 years, with no history of facial trauma or surgery, were enrolled. All subjects provided written informed consent before enrolment. Besides the earlier-mentioned age and medical history restrictions, no further selection took place. Subjects were acquired through an advertisement on a designated board at the University Medical Center Utrecht and Utrecht University.

**FIGURE 1:**
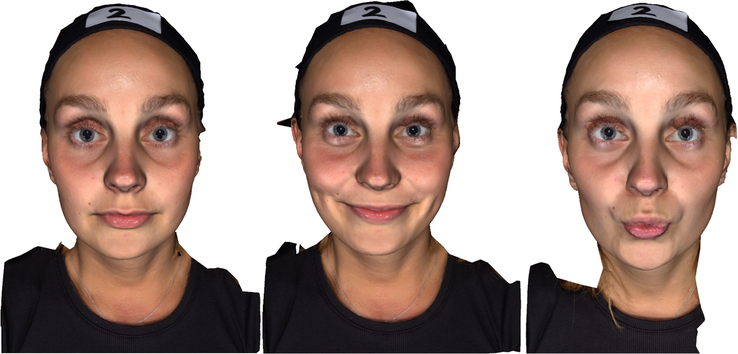
Three-dimensional photographs of the neutral face (left), closed smile (middle), and pouting (right).

A series of 3-dimensional (3D) photographs of the face were taken using the Vectra M3 (3D Imaging System; Canfield Scientific, Parsippany, NJ). Initially, a photograph was taken to capture the participant’s neutral facial position. Subsequently, participants were instructed to produce a maximum closed smile (“closed smile”) and maintain it as steady as possible. The first picture was taken at the start (*t*=0 min). Then, photographs were taken at 1-minute intervals until a duration of 6 minutes was reached (*t*=1 to *t*=6). During the whole time period, the researcher encouraged the participants for holding the facial expressions, and updating on the time left, so that participants’ thoughts would not wander too much. After a few hours break, the participants returned for the second session, where they were instructed to pout their lips (“pouting”) and the same sequence of photographs was captured. In total, each participant had 15 3D photographs taken, including one in the neutral position, 7 in the smiling position, and 7 in the pouting position. The neutral and both facial expressions are visualized in Figure 1.

### Image Processing

**FIGURE 2:**
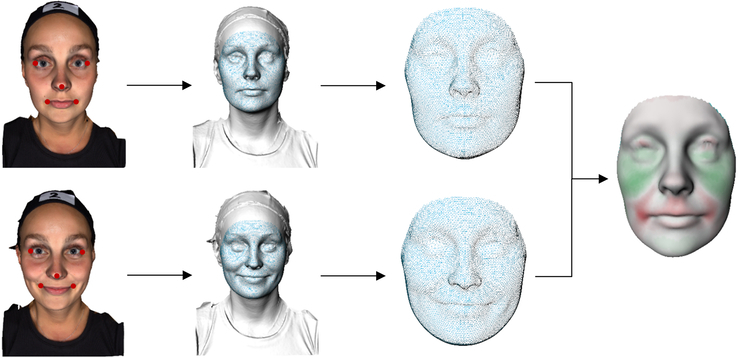
Method for facial region extraction and distance map calculation. From left to right: two 3D photographs (neutral and closed smile) were automatically annotated (red dots) followed by a registration of a facial template (in blue). The extracted facial regions are superimposed and used to calculate the distance between each corresponding vertex, resulting in a distance map. The green and red color indicate the frontal and backward differences, respectively.

The raw data were processed using the 3DMedX software package (3DMedX (v 1.2.29.3), details can be found at https://www.3dmedx.nl/). The implementation of the MeshMonk algorithm, which uses a combination of rigid and nonrigid template matching, was used to segment the facial region.^18^ A flowchart depicting the following method for facial region extraction and distance map calculation is provided in Figure 2. The algorithm uses 5 facial landmarks (exocanthions, pronasale, and cheilions) on the 3D photograph to enable rigid transformation of the template toward the 3D photograph using a simple Iterative Closest Point (ICP) algorithm. The 5 facial landmarks are automatically located using a deep-learning algorithm.^19^ Next, a regularized nonrigid deformation of the facial template toward the 3D photograph was applied, resulting in a warped and homologous 3D mesh. Using this MeshMonk algorithm for all 3D photographs resulted in a homologous data set, meaning that each vertex of each mesh corresponds with the same vertex of a second mesh, enabling comparison of the meshes in time.

To compare the 3D photographs (ie, 3D meshes) in time, distance maps were generated by superimposing two 3D meshes and calculation of the Euclidean Distance (ED) between all corresponding vertices. These ED values were used to create a colored distance map to visualize the difference between the 2 meshes. To objectively express the difference between the 2 meshes, the root mean square (RMS) was calculated using equation 1. As only a selected area of the face is of interest, a regional template was created within 3DMedX. The region of interest included the surface covering the perioral facial muscles, stretching from the bridge of the nose, over the cheeks, until the edge of the mandible. Using this template, a region-specific RMS could be extracted.


$$\mathrm{RMS}=\sqrt{\frac{1}{n}({x_{1}}^{2}+{x_{2}}^{2}+\ldots +{x_{n}}^{2})}\;(1.\;)$$

### Analyses

To investigate the duration for which a facial expression can be sustained, 3 different analyses were performed for both facial expressions. For analysis 1, each 3D photograph was compared with the 3D photograph at *t*=0 minutes to examine the change of facial expression in time. For analysis 2, each 3D photograph was compared with the previous photograph. In other words, the 3D photograph at *t*=1 was compared with the photograph at *t*=0, *t*=2 with *t*=1, and so on. A third analysis was performed, omitting the first minute, as it was suspected that most movement occurred during the first minute. Each 3D photograph was therefore compared with the photograph at *t*=1 minute. For each analysis, distance maps and RMS values were extracted for further statistical analysis.

In addition, 3 minor analyses were performed. The 3D photographs at *t*=0 of both expressions were compared with the neutral face. Second, the RMS values from analysis 1 were compared with the RMS values from analysis 3. Lastly, the RMS values from analysis 3 were also compared between the 2 facial expressions.

### Statistical Data Analysis

Statistical analysis was performed using GraphPad Prism version 8.3.0 for Windows, GraphPad Software, San Diego, Calif., www.graphpad.com. Normality was tested using Q-Q plots. Normally distributed data were expressed by means of SDs. Statistical analysis was performed using 1-tailed paired *t* tests. Statistically significant difference was considered at *P*<0.05.

## RESULTS

### Analysis 1

**FIGURE 3:**
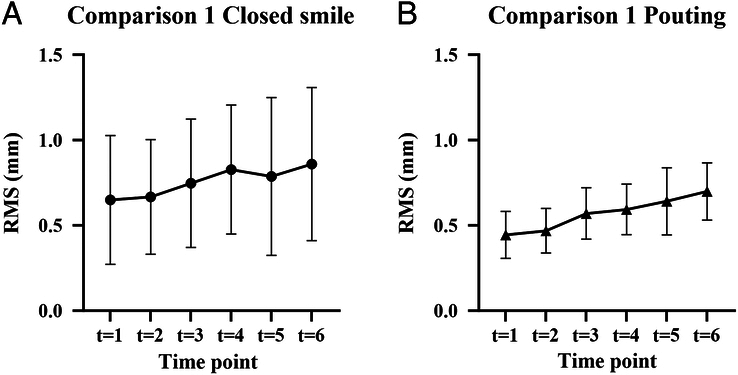
Analysis 1, comparing each image with *t*=0, mean RMS in millimeters (mm) for the closed smile (A) and pouting (B).

In this analysis, all 3D images from the series (*t*=1 to *t*=6) were compared with the 3D photo of the starting position at *t*=0. Figure 3A shows the results for the closed smile in millimeters (mm). The RMS varied between 0.23 and 1.84 mm. The mean overall RMS was 0.76±0.39 mm. Figure 3B shows the results for pouting. The RMS varied between 0.25 and 1.10 mm. The mean overall RMS was 0.57±0.17 mm.

### Analysis 2

**FIGURE 4:**
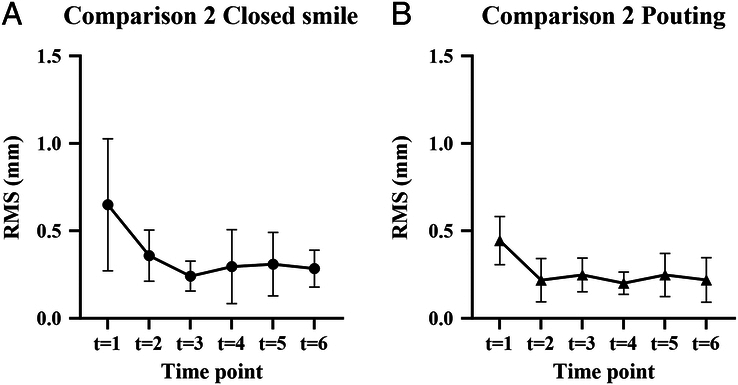
Analysis 2, comparing each image with the previous image, mean RMS in mm for the closed smile (A) and pouting (B).

In this analysis, each 3D image was compared with the previous image, meaning that t=1 was compared with *t*=0, *t*=2 to *t*=1, and so on. Figure 4A shows the results for the closed smile. The RMS varied between 0.09 and 1.31 mm. The mean overall RMS was 0.36±0.24 mm. Figure 4B shows the results for pouting. The RMS varied between 0.09 and 0.72 mm. The mean overall RMS was 0.26±0.14 mm.

### Analysis 3

**FIGURE 5:**
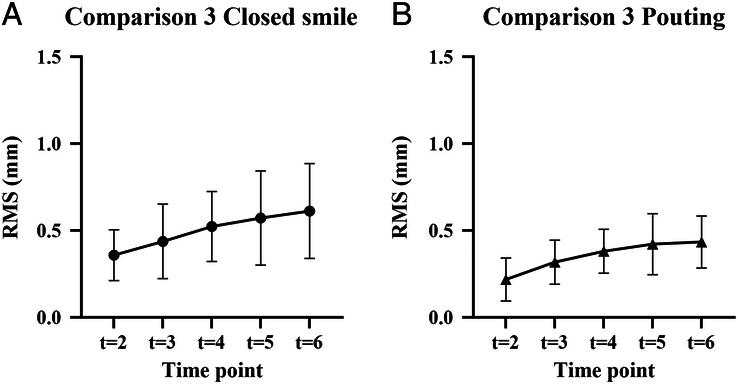
Analysis 3, comparing each image with *t*=1 (*t*=0 is excluded) for the closed smile (A) and pouting (B). Mean RMS in millimeters.

Considering that the RMS values in the first minute of the consecutive analysis are significantly higher, an additional analysis was performed. The first minute was omitted, and all images were compared with the new starting position *t*=1. Figure 5A shows the results for the closed smile. The RMS varied between 0.15 and 1.13 mm. The mean overall RMS was 0.50±0.23 mm. Figure 5b shows the results for pouting. The RMS varied between 0.09 and 0.85 mm. The results from analysis 3 were used to find differences in movement between the closed smile and pouting. The average RMS for pouting was significantly lower than RMS for smiling (0.35 versus 0.50, *P*<0.0001, 2-tailed paired *t* test).

### Minor Analyses

Comparing the image at *t*=0 compared with that of the neutral face resulted in an average RMS of 1.89 mm (SD 0.57) for the closed smile and an average RMS of 2.34 mm (SD 0.33) for the pouting face.

The next analysis, comparing the RMS values of the pouting face to those of the closed smile from analysis 3, showed significantly higher RMS values of the closed smile (0.50 versus 0.3, favoring analysis 3; *P*<0.0001, 2-tailed paired *t* test).

To compare analysis 1 and 3, results for *t*=1 were excluded from analysis 1 to enable paired *t* tests. For the closed smile, the mean difference was −0.31 (SD 0.43), with *P*<0.0001. For pouting, the mean difference was −0.23 (SD 0.26) with *P*<0.0001.

## DISCUSSION

The present study aimed to investigate the extent of movement while holding the facial expressions of a closed smile and pouting for a continuous 6-minute period using 3D imaging. To the best of our knowledge, this is the first study to specifically investigate the duration for which facial expressions, such as closed smile and pouting, can be held unchanged. The results provide valuable insights into the stability and variability of facial expressions over time, which have implications for the feasibility of capturing facial expressions under MRI. It showed that most movement occurs in the first minute, after which the facial expressions could be held relatively stable during the remaining time frame of 5 minutes. Moreover, pouting was a more stable expression to hold compared with the closed smile. Yet, pouting created a greater facial surface change than smiling.

The analyses conducted in this study revealed interesting patterns in facial movement during the 6-minute duration. Analysis 1 compared each 3D image with the initial image at *t*=0, whereas analysis 2 compared each image with the previous one, enabling a closer examination of short-term movement between consecutive time points. Analysis 3 omitted the first minute and compared all subsequent images to the new starting position at *t*=1 to assess the impact of initial movement on overall facial motion.

The results demonstrated that both the closed smile and pouting expressions showed relatively low RMS values, indicating overall stability during the 6-minute period. However, it was noteworthy that the RMS values were higher during the first minute, suggesting that initial movements significantly contribute to overall facial motion. After omitting the first minute in analysis 3, both facial expressions showed reduced RMS values, indicating increased stability after the first minute. Consequently, it is advisable to delay the start of an MRI scan until at least 1 minute has elapsed to capture a more consistent facial expression.

Several possible explanations for the observed movement within the first minute can be considered. First, the maximum facial expressions might be too demanding in terms of muscle activation and ranges of motion to be sustained for a continuous 6-minute duration. Consequently, the intensity of the facial expressions may naturally decrease after 1 minute, leading to a more stable facial expression that can be sustained relatively well over a 6-minute period. Second, participants may initially be unfamiliar with the instructions or the execution of the facial expressions, requiring time to find the optimal balance in muscle contraction and coordination. The latter can also be confirmed by a study by Lens et al,^20^ which demonstrated that movement while holding breath during MRI was highest during the first 10 seconds.

Besides the 1-minute delay between holding the expression and starting the MRI, other factors might influence stability as well. For example, placing a mirror so that the subject can see one’s own face. By enabling visual feedback, small movements are noticed quicker by the subject. This could, at least in theory, reduce movement during the scan. Whether this visual feedback actually causes the face to be held more stable has not been investigated yet. A possible downside of visual feedback might be the subject wanting to correct the attenuation and thereby actually causing movement. Another solution could be to instruct the patients to not express their maximal smile, and hold it slightly more relaxed. This would help the participants adopt a lesser intensity of the facial expression, which may help sustaining it for a longer period of time. A consequent disadvantage is that the facial expressions are not setup to the maximum, making the maximum range of motion less reproducible. This will hamper studies looking at the preoperative and postoperative putative differences in facial expressions, in surgeries like Le Fort I osteotomies, face lifts, and cleft lip repair.

The minor analyses further explored the relationship between the facial expressions and the neutral face, as well as a direct comparison between the 2 expressions. These analyses revealed interesting differences too. When comparing the expressions to the neutral face, pouting demonstrated higher RMS values compared with the closed smile when compared with the neutral face. This indicates that the facial surface changes more while pouting than while smiling. Surprisingly, when directly comparing pouting to smiling, pouting exhibited a lower mean overall RMS. This indicates that it may be a more stable expression to maintain despite being a more intense facial position, as suggested by the RMS values from the previously mentioned analysis. This result is somewhat unexpected because the existing literature had previously indicated that the closed smile was a more reproducible facial expression.^14^ Nevertheless, the present results suggest that the contraction of facial muscles during pouting might contribute to its increased stability, shedding new light on the temporal dynamics of facial expressions.

A limitation of this study is that the 3D photos were taken in a sitting position, whereas the MRI scans are usually performed with participants in the horizontal position. Unfortunately, it was not feasible to adjust the camera setup to photograph participants in a lying position. Although gravitational shifts of the skin may result in minimal changes, previous studies have indicated that these shifts are negligible.^21^ Yet, it is known that thoughts wander more significantly during the lying position compared with the sitting position.^22^ Giving relevant verbal instructions to motivate patients holding the closed smile or pouting position is considered helpful to mitigate the effect of wandering of the thoughts.^23^ Another concern that must be noted is that it is not sure whether the results of the current study are applicable to MRI. Internal muscle tremors and decreasing image quality of MRI^13^ were not directly measured. Yet, it can be assumed that these muscle tremors result in a surface change, and the best approximation to measure facial muscle tremors is through facial skin photography.

Age and sex differences were not considered in this study, as they have been found to have minimal influence on the results in previous research.^24–26^ The interval between photo captures was every other minute. This implies that movement may have occurred between photos, which were, thus, left unnoticed. The original protocol intended to capture photos every 30 seconds. But practical limitations, such as the processing time of 3D photos, hindered its implementation. In addition, it is important to note that the manual selection of 5 points in 3DMedX contained a minor error that is deemed clinically acceptable based on previous research.^27^

This study provides insights into the duration and stability of holding facial expressions for a continuous 6-minute period. The following conclusions can be drawn:

1. It is possible to maintain a facial expression for a 6-minute time period
2. The first minute should be omitted due to the amount of motion in that period
3. Pouting can be held more stable than the closed smile
4. Based on this study, MRI scanning of facial expressions seems feasible.
